# Altering the intracellular trafficking of *Necator americanus* GST-1 antigen yields novel hookworm mRNA vaccine candidates

**DOI:** 10.1371/journal.pntd.0012809

**Published:** 2025-01-10

**Authors:** Athos Silva De Oliveira, Leroy Versteeg, Neima Briggs, Rakesh Adhikari, Maria Jose Villar, JeAnna R. Redd, Peter Hotez, Maria Elena Bottazzi, Jeroen Pollet

**Affiliations:** 1 Department of Pediatrics, National School of Tropical Medicine, Baylor College of Medicine, Houston, Texas, United States of America; 2 Texas Children’s Hospital Center for Vaccine Development, Houston, Texas, United States of America; 3 Departments of Immunobiology and Internal Medicine (Infectious Diseases), Yale University, New Haven, Connecticut, United States of America; 4 Department of Biology, Baylor University, Waco, Texas, United States of America; 5 James A. Baker III Institute for Public Policy, Rice University, Houston, Texas, United States of America; Xuzhou Medical University, CHINA

## Abstract

**Background:**

The antigen *Na*-GST-1, expressed by the hookworm *Necator americanus*, plays crucial biochemical roles in parasite survival. This study explores the development of mRNA vaccine candidates based on *Na*-GST-1, building on the success of recombinant *Na*-GST-1 (r*Na*-GST-1) protein, currently assessed as a subunit vaccine candidate, which has shown promise in preclinical and clinical studies.

**Methodology/findings:**

By leveraging the flexible design of RNA vaccines and protein intracellular trafficking signal sequences, we developed three variants of *Na*-GST-1 as native (cytosolic), secretory, and plasma membrane-anchored (PM) antigens. After one immunization in mice, mRNA vaccines induced an earlier onset of antigen-specific antibodies compared to r*Na*-GST-1. Following two immunizations, mRNA vaccines induced similar or superior levels of antigen-specific antibodies compared to r*Na*-GST-1. Secretory *Na*-GST-1 was comparable to r*Na*-GST1 in producing neutralizing antibodies against *Na*-GST-1’s thiol transferase activity, while native *Na*-GST-1 induced a more robust CD8+ T cell response due to its intracellular accumulation. Although PM *Na*-GST-1 elicited one of highest titers of antigen-specific antibody and a diverse set of memory T-cell populations, it resulted in a lower ratio of neutralizing antibodies after IgG purification compared to the other vaccine candidates.

**Conclusions/significance:**

These findings emphasize the importance of antigen localization in tailoring immune responses and suggest that extracellular antigens are more effective for inducing humoral responses, whereas cytosolic antigen accumulation enhances MHC-1 peptide presentation. Future studies will determine if these *in vitro* and immunogenicity findings translate to *in vivo* efficacy. Altogether, mRNA vaccines offer numerous possibilities in the development of multivalent vaccines with single or multiple antigens.

## Author summary

Our work on *Na*-GST-1 mRNA vaccines addresses a critical need in the fight against hookworm infections, which affect more than 100 million people worldwide. *Na*-GST-1, a protein essential for the survival of hookworms, has been explored as a target for vaccine development. By using mRNA technology, we designed vaccine candidates that express and deliver *Na*-GST-1 to different locations in cells and tissues of immunized mice, including forms that are secreted, anchored to the plasma membrane, or retained within cells (the native form). Our findings reveal that the location of antigens within cells significantly impacts the immune response. Secreted and membrane-anchored forms of *Na*-GST-1 generated strong antibody responses, which are important for neutralizing *Na*-GST-1 in the hookworm. Meanwhile, the intracellular and membrane anchored forms elicited a robust cytotoxic cell response, which would be more important for targeting intracellular pathogens. This research not only advances our understanding of how protein localization affects immune responses but also highlights the versatility and potential of mRNA vaccines to target various pathogens by manipulating how and where vaccine antigens are presented to the immune system.

## Introduction

As a leading cause of non-dietary iron-deficiency anemia worldwide, hookworm infection can hinder physical and cognitive development [1,2]. Primarily acquired through skin contact with contaminated soil, these parasitic infections mostly occur in tropical and subtropical regions of the globe with limited sanitation infrastructure [3]. More than 100 million people are infected with hookworms (https://www.healthdata.org/), and the associated disease is becoming dominant in parts of Africa and Southeast Asia affected by climate change [4]. Despite the effectiveness of benzimidazole anthelmintics, reinfections can promptly occur post-treatment, with a concern of drug-resistant parasites emerging [5,6]. Recognizing the limitations of current treatments, vaccines can be a comprehensive solution by providing long-lasting protection, either as isolated interventions or in combination with other measures. However, developing a vaccine has been challenging due to the nature of hookworms as large extracellular parasites with intricate life cycles, and no commercially available helminth vaccine as a basis for modeling [7]. Additionally, these organisms have evolved sophisticated mechanisms to manipulate the human immune system to prevent acquired immunity, allowing them to survive for years within the host, often leading to reinfection with a similar or even greater disease burden [8–10].

The term hookworm encompasses multiple nematode species within the family Ancylostomatidae notable for their mouthpart teeth or cutting plates [11,12]. These structures serve to attach the worms to the gut of vertebrate hosts, facilitating the rupture of blood vessels and leading to blood extravasation. The adult hookworms then ingest the blood relying on host hemoglobin as a nutrient source [13]. *Necator americanus* is the most prevalent and widely distributed human hookworm, making it the prime focus of vaccine research that is currently advancing through clinical trials [14,15]. Apart from a few studies using live attenuated worms or larvae, specific antigens crucial for parasite survival and host interaction have been targeted in vaccine programs since the 1980s [16–19].

The initial lead hookworm vaccine antigen, the larvae entry antigen *N*. *americanus* Ancylostoma-secreted protein 2 (*Na*-ASP-2), was discontinued in clinical trials due to an urticarial reaction in some adults from a hookworm endemic region of Brazil, who were later found to have pre-formed anti-*Na*-ASP-2 IgE [20]. Second-generation vaccine candidates focused on antigens involved in hookworm blood feeding, resulting in the selection of *N*. *americanus* aspartic-protease-1 (*Na*-APR-1) and *N*. *americanus* glutathione-S-transferase-1 (*Na*-GST-1). These candidates were chosen after showing protection efficacy in preclinical studies and after serum screening within hookworm-endemic populations to confirm the absence of antigen-specific IgE. *Na*-APR-1 and *Na*-GST-1 are enzymes that play key roles in parasite blood feeding and hemoglobin digestion and heme binding or detoxification, respectively [13,21,22]. *Na*-APR-1 is an aspartic protease, while *Na*-GST-1 belongs to a specific Nu class of glutathione S-transferases with a large pocket to bind ligands [23,24]. Vaccine-induced antibodies that bind to these enzymes can inhibit their function, interfering with the worm’s blood feeding. Vaccination with recombinant *Na*-APR-1 and *Na*-GST-1 protein has been demonstrated to effectively reduce worm burdens in different animal models [25–28]. Both recombinant protein antigens are being accelerated in clinical trials conducted in adults and children and have been shown to induce robust antibody responses and T-cell responses [26,29,30]. *Na-*GST-1 offers the benefit of its high-yield and low-cost expression in a *Pichia pastoris* yeast system, making it an attractive lead vaccine candidate for global health and resource-poor health systems [31]. Our Texas Children’s Hospital Center for Vaccine Development has previously employed this approach to develop a subunit COVID-19 vaccine technology that resulted in the administration of approximately 100 million doses in India and Indonesia [32].

Although the current generation hookworm protein subunit vaccine formulations provided significant immunogenicity in humans and protective immunity in laboratory animals (with human challenge studies pending), there is an interest in exploring alternative antigen delivery systems. In this context, RNA technology offers a standardized method for rapid design and production of vaccine candidates [33]. By simply altering the genetic code within mRNA, the same antigen can be translated into multiple versions in recipient cells and tissues [34]. Introducing signal sequences to the mRNA can further alter antigen trafficking in transfected cells, influencing the overall immune response [34–36]. Crucially, these modifications overcome limitations associated with subunit vaccines, such as challenges in solubility, purification, and formulation, seen during the production of recombinant proteins in heterologous systems.

RNA and subunit vaccines can elicit distinct immune responses due to the differences in the spatial and temporal availability of antigens [37]. Immediately after subunit vaccine immunization, antigen-presenting cells (APCs) endocytose free-protein antigens, processing them into peptides presented by major histocompatibility complex (MHC) class II to CD4^+^ T lymphocytes [38]. In RNA vaccines, recipient cells first translate RNA into protein, a process that occurs intracellularly. While certain antigens are naturally secretory or transmembrane, becoming readily accessible to APCs after translation, others predominantly accumulate in the cytosol. In the cytosol, these antigens can undergo degradation by the proteasome into peptides, subsequently loaded into MHC class I for presentation to CD8^+^ T lymphocytes [34,36]. Consequently, mRNA vaccines encoding cytosolic antigens may trigger a more robust CD8^+^ T cell response compared to extracellular antigens collected and processed by APCs and presented to CD4^+^ T cell response via MHC class II [39]. Protection mediated by immunization with *Na*-APR-1 and *Na*-GST-1 is thought to rely primarily on antibody-mediated neutralization of these enzymes, as shown with *Na*-APR-1 [40]. Thus, an ideal CD4^+^ T cell response would induce B cell activation, proliferation, and differentiation into antigen-specific plasma cells and memory B cells [41].

Leveraging the advantages of RNA technology, three mRNA vaccine candidates were strategically designed to direct the accumulation of *Na*-GST-1 in the cytosol, to be secreted, or to be anchored in the plasma membrane (PM) of transfected cells. Following immunization of BALB/c mice, both antigen-specific antibody titers and cellular response of splenocytes were quantified to evaluate the immune response to the three mRNA vaccine candidates, also in comparison with the well-studied recombinant *Na*-GST-1 produced in *Pichia pastoris* [22]. Our findings not only highlight the versatility of mRNA vaccines in addressing helminth infections, but also reinforce that we can intentionally alter antigen trafficking and presentation to shape an immune response against a specific pathogen.

## Material and methods

### Ethics statement

Immunization and sampling procedures strictly adhered to the Guide for the Care and Use of Laboratory Animals [46]. The protocol under number AN-5765 was approved by the Institutional Animal Care and Use Committee (IACUC) at Baylor College of Medicine.

### RNA production

Human codon-optimized *Na-gst-1* gene (GenBank: FJ711440) was synthesized by Twist Biosciences (San Francisco, CA) and subsequently cloned into plasmid backbones containing, from 5’ to 3’, an *AG* T7 promoter (CleanCap compatible), 5’ untranslated region (UTR), Kozak sequence, cloning site, 3’ UTR, and segmented poly(A) tail [42]. To generate secretory *Na*-GST-1, the plasmid also included an IgG signal peptide sequence upstream of the cloning site [43]. For anchored *Na*-GST-1, the plasmid contained albumin signal peptide and CD55 GPI attachment sequences upstream and downstream of the cloning site [44]. Additionally, a FLAG tag (DYKDDDDK) coding sequence was added to the 5’ (following the signal peptide) for anchored *Na*-GST-1, and to the 3’ end for the other constructs. Plasmids were then linearized and utilized as template for *in-vitro* transcription reactions (co-transcription capping) following the protocol of CleanCap Reagent M6 (Trilink BioTechnologies, San Diego, CA), with rUTP substituted for m^1^ΨTP [45]. After DNAse I treatment, mRNA was purified using Monarch Spin RNA Cleanup Kit (NEB, Ipswich, MA). To assess size and integrity, mRNA was heated at 70°C for 10 min before loading into a 1.5% agarose gel.

### *In vitro* RNA transfection and protein localization

#### Cell culture

DC2.4 murine dendritic cell line was maintained in RPMI 1640+L-glutamine supplemented with 10% fetal bovine serum (FBS), antibiotics, 1 mM non-essential amino acids, 10 mM HEPES, and 55 μM beta-mercaptoethanol under 5% CO_2_ at 37°C.

#### Immunocytochemistry (IC)

Around 1.2E5 DC2.4 cells were seeded per well in a 24-well plate. The next day, transfections were conducted by mixing 500 ng mRNA with Lipofectamine MessengerMAX [Thermo Fisher Scientific (TFS), Waltham, MA], following the manufacturer’s instructions. After 20 hours, cells were either fixed or fixed and permeabilized using Cytofix fixation buffer or Cytofix/Cytoperm fixation/permeabilization buffer (BD, Franklin Lakes, NJ) for 30 min at 4°C. Cells were washed three times with staining buffer [2% FBS in phosphate buffered saline (PBS) for only-fixed cells] or Perm/Wash buffer (BD) and then incubated with anti-FLAG monoclonal antibody conjugated with Alexa Fluor 488 (TFS, Cat. MA1-142-A488) for 60 min at 4°C. The antibody was diluted 1:250 in either staining buffer or Perm/Wash buffer. Following a second wash step, images were captured using an inverted fluorescence microscope.

#### Western blot (WB) analysis for FLAG tag detection

About 2.5E5 DC2.4 cells were seeded per well in a 12-well plate and transfected with 1 μg mRNA-Lipofectamine MessengerMAX complexes on the following day. After a 20-hour incubation period, cells were washed once with PBS and detached using cell dissociation reagent Accutase (MilliporeSigma, Burlington, MA). The harvested cells were resuspended with 50 μl RIPA buffer and incubated on ice for 30 min with agitation. Subsequently, the samples were centrifuged at 13.000 x g for 20 min. After quantification with BCA Assay (TFS), 10 μg total protein per sample were loaded onto a 4–12% Bis-Tris SDS-PAGE gel. Monoclonal anti-FLAG M2 (MilliporeSigma, Cat. F3165, 1:1000) was used as primary antibody, and alkaline phosphatase goat anti-mouse (KPL, Cat. 5220–0357, 1:3000) as secondary antibody. Washing steps were performed with PBST (0.05% Tween-20 in PBS), and detection was carried out using NBT/BCIP substrate.

#### FLAG tag immunostaining and flow cytometry

After transfection in a 12-well plate, cells were detached, washed with PBS, and counted. Around 1.0E5 cells were seeded per well in a laminar wash 96-well plate (Curiox Biosystems, Seoul, South Korea) and incubated for 20 min at 4°C. Once settled, cells underwent ten wash cycles with staining buffer (2% FBS in PBS) using a Laminar Wash HT2000 (Curiox Biosystems) for all washing steps. The cells were then resuspended in 70 μl Cytofix/Cytoperm buffer and incubated for 20 min at 4°C, followed by 10 wash cycles with Perm/Wash buffer. Subsequently, cells were resuspended in 25 μl Cytofix/Cytoperm buffer containing 1 μl mouse Fc block (BD) and incubated for 5 min at 4°C. Immediately after, 45 μl Cytofix/Cytoperm buffer containing anti-FLAG M2-Cy3 (MilliporeSigma, Cat. A9594, 1:100) was added to the cells which were incubated for more 30 min at 4°C, followed by a final wash step of 15 cycles. Finally, cells were analyzed for FLAG staining using a Guava Muse Flow Cytometer (Cytek Biosciences, Fremont, CA).

#### RNA formulation into lipid nanoparticles (LNPs)

*Na*-GST-1 mRNAs were formulated with Genvoy ILM lipid reagent (Precision Nanosystems, Cat. NWW0042) at a nitrogen-to-phosphate ratio of 4:1 using a NanoAssemblr Ignite instrument (Precision Nanosystems). The resulting mRNA/LNP complexes were concentrated using 30 kDa spin filter columns and sterilized through 0.2 μm disk filters. The average LNP size was 90 nm with a polydispersity index of less than 15%, as determined by dynamic light scattering using a Zetasizer Nano ZS90 (Malvern Panalytical, UK). The mRNA concentration was measured using a RiboGreen RNA Assay kit (Thermo Fisher Scientific, USA Cat. R11490). Experiments with and without TritonX100 detergent indicated that the loading efficiency of the encapsulated mRNA was over 85% for all mRNA LNP formulations. In a final step, mRNA/LNP vaccine formulations were diluted to a concentration of 200 μg/ml mRNA in sterile PBS with 8% sucrose and stored at -80°C until use.

### Recombinant *Na*-GST-1 protein vaccine

*Na*-GST-1 protein was produced in *Pichia pastoris* and purified as published elsewhere [22]. The protein was adsorbed to aluminum hydroxide adjuvant (Alhydrogel, Croda Denmark) in a glucose imidazole buffer (10% glucose, 10 mM imidazole, pH 7.4).

### Immunogenicity study

#### Immunization

Forty female BALB/c mice (cAnNTac, Taconic Biosciences), aged 6–8 weeks, were divided into 5 groups, each consisting of 8 individuals. Two intramuscular immunizations were administered three weeks apart (switching leg for the second injection), followed by euthanasia at day 42. Each group received either placebo (empty LNPs), 10 μg mRNA/LNPs, or 20 μg *Na*-GST-1 protein with 160 μg of aluminum hydroxide. For serum collection, blood samples were obtained on immunization days and at the time of euthanasia. Fecal pellets from the large intestine and spleens were collected after euthanasia. Splenocytes were processed for flow cytometry as published elsewhere [47].

#### ELISA

Ninety-six-well flat bottom plates were coated overnight at 4°C with 100 μl of 0.25 μg/ml r*Na*-GST-1 diluted in KPL coating solution (SeraCare Life Sciences, Milford, MA), followed by blocking with 200 μl dilution buffer (0.1% BSA in PBST) for two hours at room temperature. After a single wash with PBST, wells were incubated with 100 μl of diluted sera in duplicates for two hours at room temperature. Mouse sera were serially diluted three-fold, ranging from 1:200 to 1:437,400. Naïve mouse sera were also included in all plates, serving as the cutoff. After incubation, plates were washed four times and incubated with 100μl of either goat anti-mouse IgG HRP, goat anti-mouse IgG1 HRP or goat anti-mouse IgG2 HRP (Lifespan Bioscience, Shirley, MA) in dilution buffer for one hour at room temperature. After five washes, wells were incubated with 100 μl TMB substrate for 15 min. Reactions were stopped with 100 μl 1M HCl, and absorbance was measured at 450 nm using a BioTek Epoch 2 spectrophotometer (Agilent, Santa Clara, CA). For data analysis, duplicates were averaged, and titers were calculated using a four-parameter logistic regression curve. Titer cutoff values were determined by adding the average of naïve mouse control to three times its standard deviation.

To perform fecal IgA and IgG ELISA, pellets were solubilized in extraction buffer (10% goat serum in PBS) at a ratio of 100 μl buffer per 10 mg pellet, followed by vortexing until complete disruption. Samples were clarified by centrifugation at 13,000 g for 10 min. For ELISA, fecal samples were serially diluted two-fold, ranging from 1:8 to 1:1024. In addition to goat anti-mouse IgG HRP, samples were incubated with goat anti-mouse IgA HRP (Southern Biotech, Birmingham, AL).

#### Splenocyte immunostaining and flow cytometry

Around 1.0E6 splenocytes per mouse were seeded per well into 96-well plates. Cells were subjected to three conditions (in cRPMI medium): restimulated with 10 μg/ml *Na*-GST-1, stimulated with PMA/I (positive stimulation control), or unstimulated. Plates were incubated for 48 hours at 5% CO_2_ and 37°C, with Brefeldin A (BD) added during the final five hours of incubation. Subsequently, the cells were transferred to a laminar wash 96-well plate, and all wash cycles were executed using a Laminar Wash HT2000. Once settled on the plate, cells underwent 10 wash cycles with 1X PBS and resuspended in viability dye-containing 1X PBS. After 30 min incubation at 4°C, cells underwent 10 wash cycles with staining buffer. CD16/CD32 Fc receptors were blocked with 2 μl mouse Fc Block (BD), followed by five-minute incubation before addition of the surface marker antibody cocktail (CD3, CD4, CD8, CD25, CD44, and CD62L) and a further incubation of 30 min at 4°C. After 10 wash cycles, cells were resuspended in Cytofix/Cytoperm buffer and incubated for 20 min at 4°C, followed by 10 additional wash cycles with Perm/Wash buffer. Subsequently, an intracellular marker antibody cocktail (IL-2, IL-4, IL-13, IFN-γ, TNF-α, and granzyme B) was added. Following incubation for 30 min at 4°C, cells underwent 15 wash cycles before being transferred to 96-well culture plates. Samples were finally analyzed using an Aurora Spectral Flow Cytometer (Cytek). Single-stained cells and bead controls were used to unmix the raw data. Fluorescence minus one (FMO) of the samples and untreated controls were used to gate the cell populations using FlowJo software. The percentages of unstimulated cell populations were subtracted from the values of *Na*-GST-1 stimulated cells to obtain the results.

#### Statistical analysis

Statistical analysis was conducted using GraphPad Prism software. The Kruskal-Wallis test was performed initially to assess overall differences among groups, followed by Dunn’s test for multiple comparisons to identify specific pairwise differences. For flow cytometry data, p-values were corrected for multiple comparisons to control Type I error. In contrast, ELISA data were analyzed using uncorrected p-values to prioritize the detection of biologically relevant differences and to mitigate the risk of Type II errors (false negatives), which were observed when corrections were applied. Data are presented as mean ± standard deviation, with differences considered statistically significant at p < 0.05.

#### Neutralization assay of *Na*-GST-1 thiol transferase activity

Total IgG was purified from pooled sera of each immunization group using Nab Protein G spin columns (TFS). The neutralization assay of *Na*-GST-1 thiol-transferase activity was performed using the GST Fluorometric Activity Assay Kit (Abcam, Cambridge, UK) following the manufacturer’s protocol, but including modifications as published elsewhere [48]. Specifically, three concentrations of purified IgG (10, 5, and 2.5 μg in a 10 μl volume) were incubated in triplicates with 0.225 μg *Na*-GST-1 protein (in 90 μl GST Assay Buffer) in a 96-well black flat bottom plate for 1 hour at 37°C with agitation. Then, 10 μl of glutathione followed by 100 μl of monochlorobimane (MCB) solution was added per well. Fluorescence was measured at Ex/Em = 380/460 nm in kinetic mode every 5 min for 1 hour. The time point of 20 min was selected within the linear range to calculate *Na*-GST-1 activity.

## Results

### Signal sequences alter the intracellular trafficking of *Na*-GST-1

**Fig 1:**
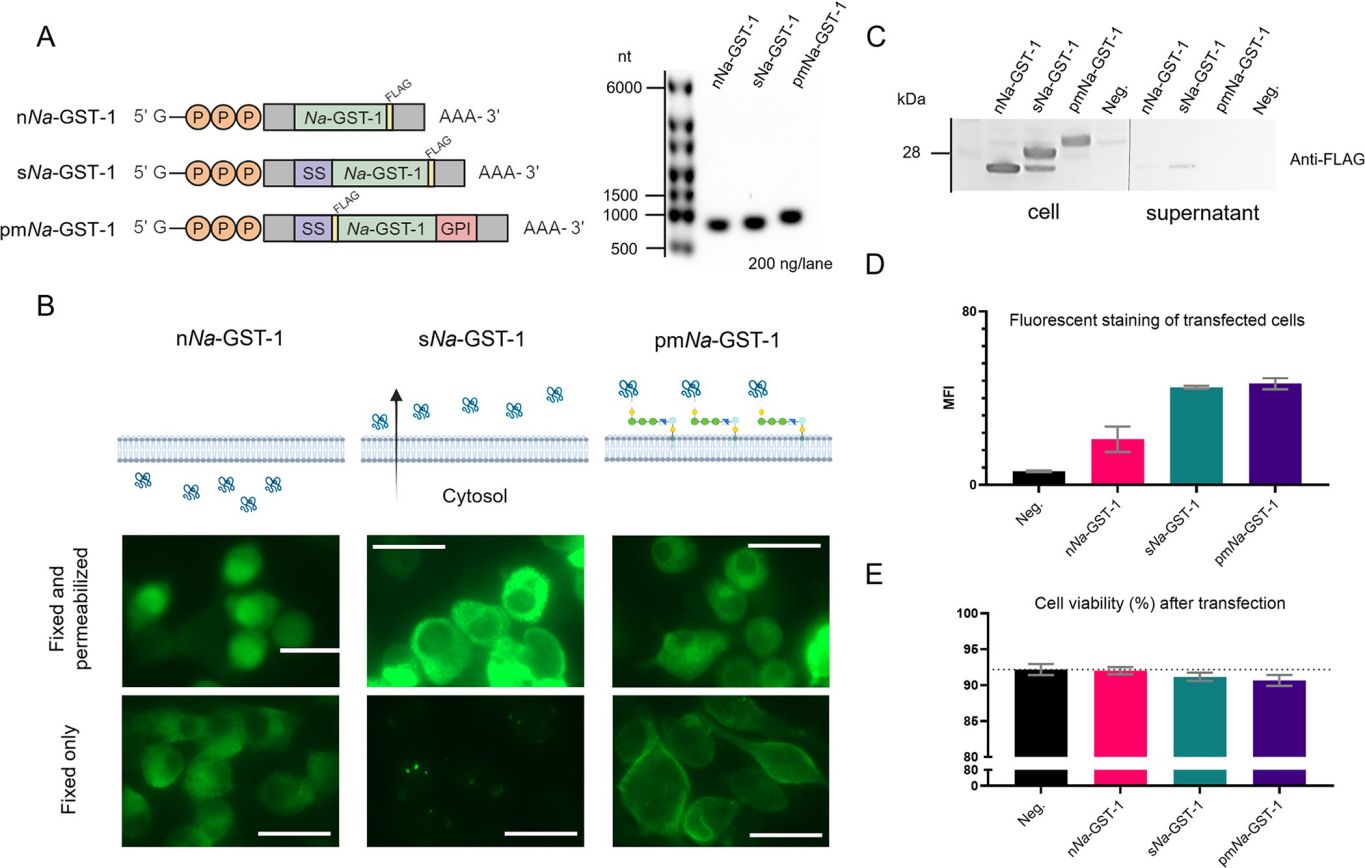
*In vitro* validation of mRNAs encoding different forms of *Na*-GST-1. (A) Schematic of *Na*-GST-1 mRNA vaccine candidates—native (n), secretory (s), and plasma membrane-anchored (pm)—and their migration in a 1.5% agarose gel. In the diagram, "SS" represents the signal peptide sequence, and "GPI" denotes the glycosylphosphatidylinositol attachment sequence. Gray regions indicate the 5’ and 3’ UTRs, while "PPP" marks the triphosphate linkage connecting the 7-methylguanosine cap to the first base of the mRNA. Schematic created with Biorender.com. (B) Immunocytochemistry of transfected DC2.4 cells. Images were captured 20 hours post-transfection. Alexa Fluor 488-conjugated mouse anti-FLAG was used for immunostaining. Cells were both fixed and permeabilized for immunostaining within membrane-bound organelles or only fixed for immunostaining in the cytosol and PM. Scale bars represent 25 μm. Schematics depict expected protein localization in each case. (C) WB analysis of cell pellets and supernatants from transfected DC2.4 cells. (D) Flow cytometry analysis of transfected DC2.4 cells immunostained with Cy3 conjugated mouse anti-FLAG. MFI: Median fluorescence intensity. (E) Cell count and viability of transfected DC2.4 cells. The dotted line indicates the average cell viability level of the untreated cells (Neg.).

*Na*-GST-1, comprising 206 amino acids with a molecular weight of 23.68 kDa, does not contain a predicted signal peptide according to the SignalP 6.0 server [49]. This suggests that *Na*-GST-1 predominantly accumulates in the cytosol. Based on this premise, three mRNA vaccine candidates for *Na*-GST-1 were developed in-house, encoding native *Na*-GST-1 (n*Na*-GST-1), secretory *Na*-GST-1 (s*Na*-GST-1), and PM-anchored *Na*-GST-1 (pm*Na*-GST-1). s*Na*-GST-1 included a signal peptide for endoplasmic reticulum (ER) import and secretion, while pm*Na*-GST-1 contained both a signal peptide for ER import and a GPI attachment sequence for PM anchoring. These mRNAs were successfully generated by co-capping *in-vitro* transcription (Fig 1A).

To determine the site of antigen trafficking post-translation, two distinct IC protocols were applied to transfected DC2.4 mouse dendritic cells. The first protocol involved cell fixation-and-permeabilization, allowing for immunostaining of FLAG-tagged *Na*-GST-1 within membrane-bound organelles. In contrast, the second protocol involved only cell fixation, restricting immunostaining to the cytosol and PM due to the inability of antibodies to cross internal organelle membranes of DC2.4 cells. Analysis revealed that n*Na*-GST-1 exhibited a nucleocytoplasmic distribution, easily visible in fixed-and-permeabilized cells (Fig 1B). Differently, s*Na*-GST-1, which contains an ER import signal peptide, is localized in the endomembrane system, as evidenced by its absence in the cytosol of cells that underwent only fixation. Secretion of s*Na*-GST-1 into the supernatant was also confirmed by WB (Fig 1C). Furthermore, pm*Na*-GST-1 was successfully anchored to the PM, covering the outer layer of transfected cells (Fig 1B).

Given the variations in translation efficiency observed in IC images among the three mRNA candidates, cells were immunostained after fixation-and-permeabilization, followed by flow cytometry analysis to measure protein expression. Consistent with IC results, the median fluorescence intensity of cells expressing s*Na*-GST-1 and pm*Na*-GST-1 was higher compared to cells expressing n*Na*-GST-1 (Fig 1D). It is important to note, however, that the spatial distribution of the different antigens could also have influenced the measured fluorescent signal. For example, pm*Na*-GST-1 is thought to be more exposed to antibody binding during immunostaining, potentially leading to a higher fluorescence intensity. Regardless of translation efficiency, all DC2.4 cells transfected with mRNAs exhibited comparable viability to cells treated only with the transfection reagent, suggesting that the different forms of *Na*-GST-1 are non-toxic protein products (Fig 1E).

### s*Na*-GST-1 and pm*Na*-GST-1 boost the production of antigen-specific IgG

**Fig 2:**
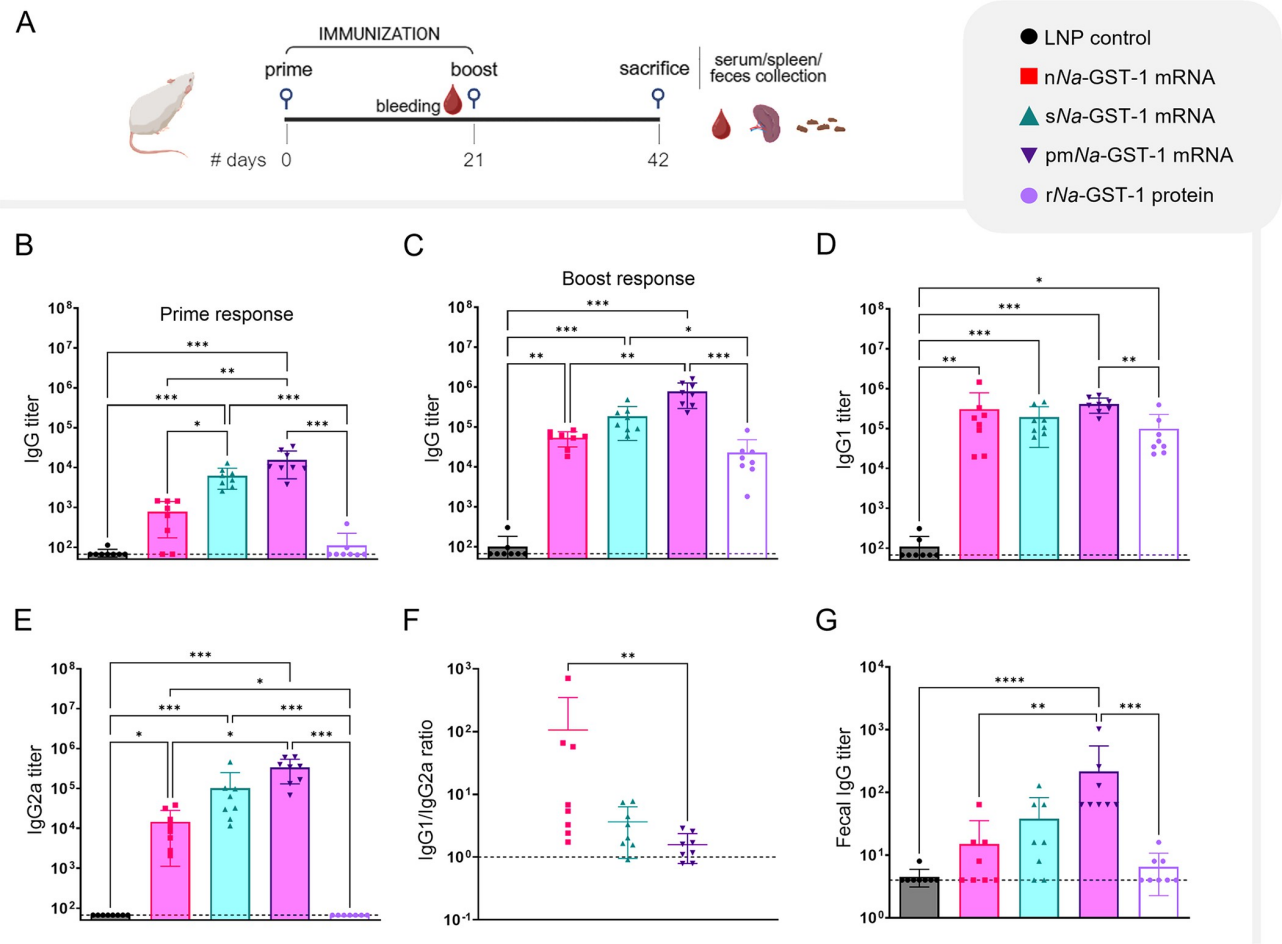
Antigen-specific IgG titers induced by *Na*-GST-1 mRNA vaccine candidates. (A) Immunization schedule for BALB/C mice with timeline for sample collection. Schematic created with Biorender.com. (B-E) *Na*-GST-1-specific total IgG and IgG subclass titers in mouse sera measured by indirect ELISA. IgG subclasses were assessed only for sera collected after the second immunization (day 42). (F) IgG1:IgG2 ratio for groups that showed induction of both IgG subclasses. (G) *Na*-GST-1-specific total IgG measured in mouse feces by indirect ELISA. In all panels, except for (F), the dashed line indicates the titer cutoff. Each data point represents the average of technical duplicates. Statistical analysis was performed using the Kruskal-Wallis test, followed by Dunn’s test for pairwise comparison of groups. *p < 0.05, ** p < 0.01, ***p < 0.001, ****p < 0.0001.

After *in vitro* validation, mRNAs were encapsulated in LNPs for immunization in mice. Forty mice were divided into five groups, each comprising eight individuals, and immunized twice (Fig 2A). Two control groups received either recombinant *Na*-GST-1 protein produced in *P*. *pastoris* (r*Na*-GST-1) or empty LNPs. Twenty-one days after the first immunization dose, s*Na*-GST-1 and pm*Na*-GST-1 induced higher levels of serum antigen-specific IgG titers compared to the other three groups (Fig 2B). Following the second dose, IgG titers in n*Na*-GST-1 group reached comparable levels with r*Na*-GST-1. The group immunized with pm*Na*-GST-1 continued to show a higher titer in antigen-specific IgG titers compared to both n*Na*-GST-1 and r*Na*-GST-1 (Fig 2C).

The role of immunoglobulin class or subclass in the response to *Na*-GST-1 remains unclear. However, in line with previous studies, we measured serum antigen-specific IgG1 and IgG2a subclasses, as crude correlates to T helper 2 (Th2) and T helper 1 (Th1) cells [22,50,51]. Significant levels of IgG1 and IgG2a were observed across all mRNA groups compared to the empty LNP control (Fig 2D). An increase in IgG1 was seen for pm*Na*-GST-1 compared to r*Na*-GST-1, while IgG2a was only induced in the mRNA groups, with pm*Na*-GST-1 inducing higher titers than n*Na*-GST-1 (Fig 2E). IgG1 to IgG2a ratio suggested that pm*Na*-GST-1 induces the most balanced IgG response (Fig 2F).

Total IgG was also measured in fecal samples collected from the large intestine of mice. Elevated titers of antigen-specific IgG were observed only in the pm*Na*-GST-1 group (Fig 2G). No differences in antigen-specific IgA levels were detected among any vaccination groups compared to the control.

### pm*Na*-GST-1 mRNA induces the most diverse set of T cell populations

**Fig 3:**
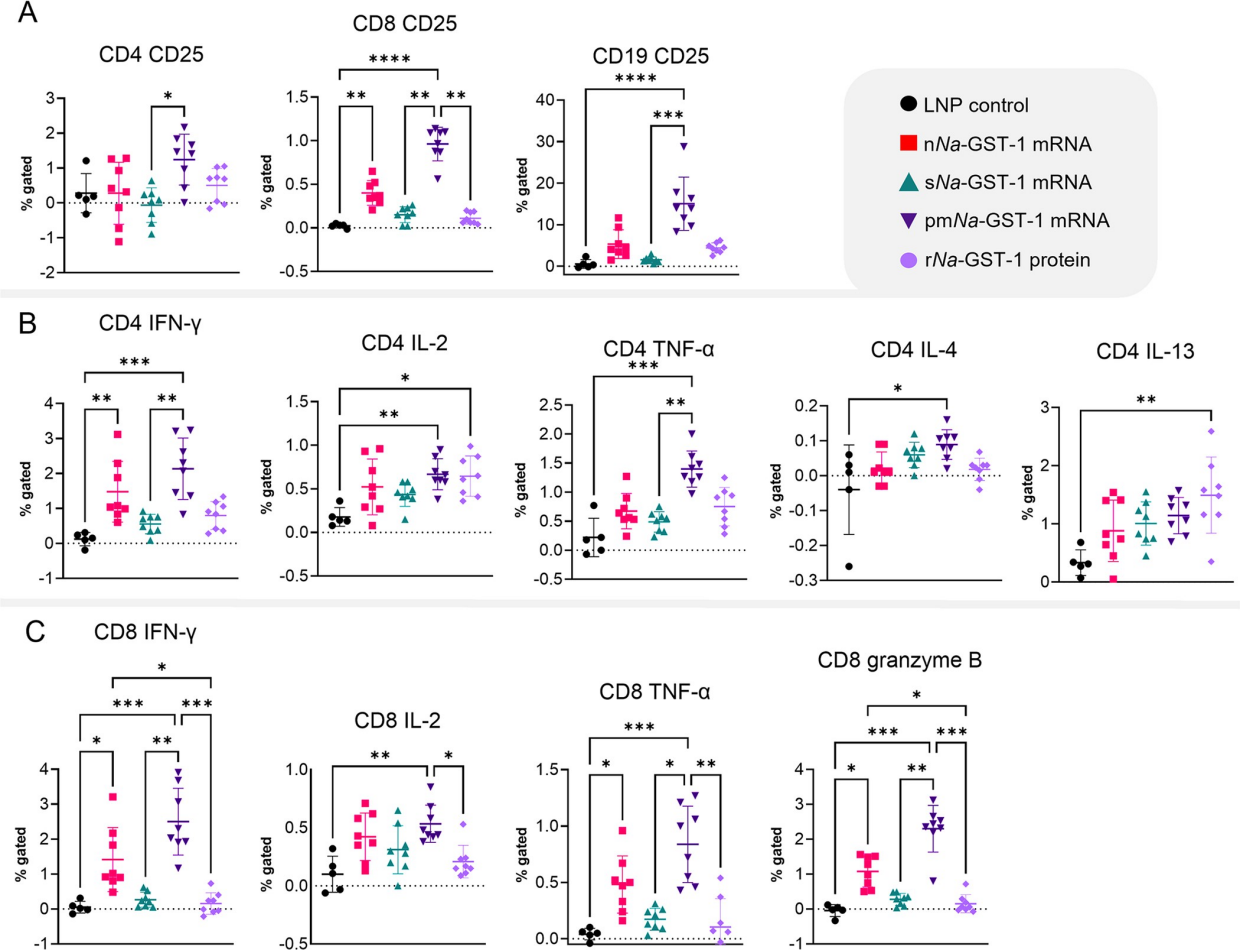
Percentage of CD25-expressing lymphocytes and cytokine-producing T cells after splenocyte restimulation with r*Na*-GST-1 protein. (A) Percentage of CD4+, CD8+, and CD19+ lymphocytes expressing CD25 after antigen restimulation. (B) Percentage of CD4+ cells producing cytokines following antigen restimulation. (C) Percentage of CD8+ cells producing cytokines following antigen restimulation. Statistical analysis was performed using the Kruskal-Wallis test, followed by Dunn’s test for pairwise comparison of groups. *p < 0.05, ** p < 0.01, ***p < 0.001.

To investigate the antigen-specific T-cell populations elicited after immunization, splenocytes were cultured and restimulated with r*Na*-GST-1 protein, followed by flow cytometry analysis. CD25 was used as an indicator of antigen-specific activation for CD4+ and CD8+ T cells, and as a marker of maturation and efficient antigen presentation for B cells [43,44]. While CD25-expressing CD8+ T cells increased in the groups immunized with n*Na*-GST-1 and pm*Na*-GST-1 mRNAs, no significant differences were observed for CD4+ T cells when comparing the vaccine groups and the LNP control. Additionally, an increase in CD25+ B cells was observed only for the pm*Na*-GST-1 group (Fig 3A).

For cytokine-producing CD4+ T cells, higher expression of IFN-γ was observed in the n*Na*-GST-1 and pm*Na*-GST-1 groups, indicating a strong Th1 response (Fig 3B). In the pm*Na*-GST-1 group, expressions of TNF-α, IL-2, and IL-4 were also up-regulated, suggesting a mixed Th1/Th2 response. An increase in IL-13 was only observed in the r*Na*-GST-1 group. Similar results were also seen in memory and effector CD4+ T cells (S1 Fig). Overall, pm*Na*-GST-1 mRNA was particularly more effective in inducing a robust and diverse cellular response, which does not seem to be directly associated with humoral response, as s*Na*-GST-1 mRNA induced high antibody titers.

Increased expression of IFN-γ and TNF-α was also observed in CD8+ T cells from both n*Na*-GST-1 and pm*Na*-GST-1 groups, with IL-2 also elevated in the latter (Fig 3C). Furthermore, when examining central memory CD8+ cells, upregulation of these three cytokines was observed in the n*Na*-GST-1 and pm*Na*-GST-1 groups, while IFN-γ was specifically increased in pm*Na*-GST-1 when analyzing effector memory CD8+ cells (S2 Fig). Importantly, granzyme B expression was also increased, reinforcing that the intracellular accumulation and anchoring of *Na*-GST-1 enhances T cytotoxic cellular responses.

### mRNA vaccine candidates induce neutralizing antibodies against *Na*-GST-1

Protection by targeting *Na*-GST-1 is thought to be mediated by antigen-specific antibodies that neutralize the capacity of this enzyme in detoxifying pro-oxidants generated during digestion of host hemoglobin by hookworms. To evaluate the effectiveness of mRNA vaccine-induced antibodies in this context, the inhibition of glutathione-S-transferase activity of *Na*-GST-1 was assessed *in vitro* using a commercial fluorometric GST assay kit with modifications, as published elsewhere [48]. The assay was inconsistent when using whole serum. Thus, purified IgG was used as in previous publications [48]. For each group, purified IgG from pooled sera was mixed with a fixed amount of recombinant *Na*-GST-1 protein (0.225 μg), followed by the addition of glutathione and a fluorescent thiol substrate.

**Fig 4:**
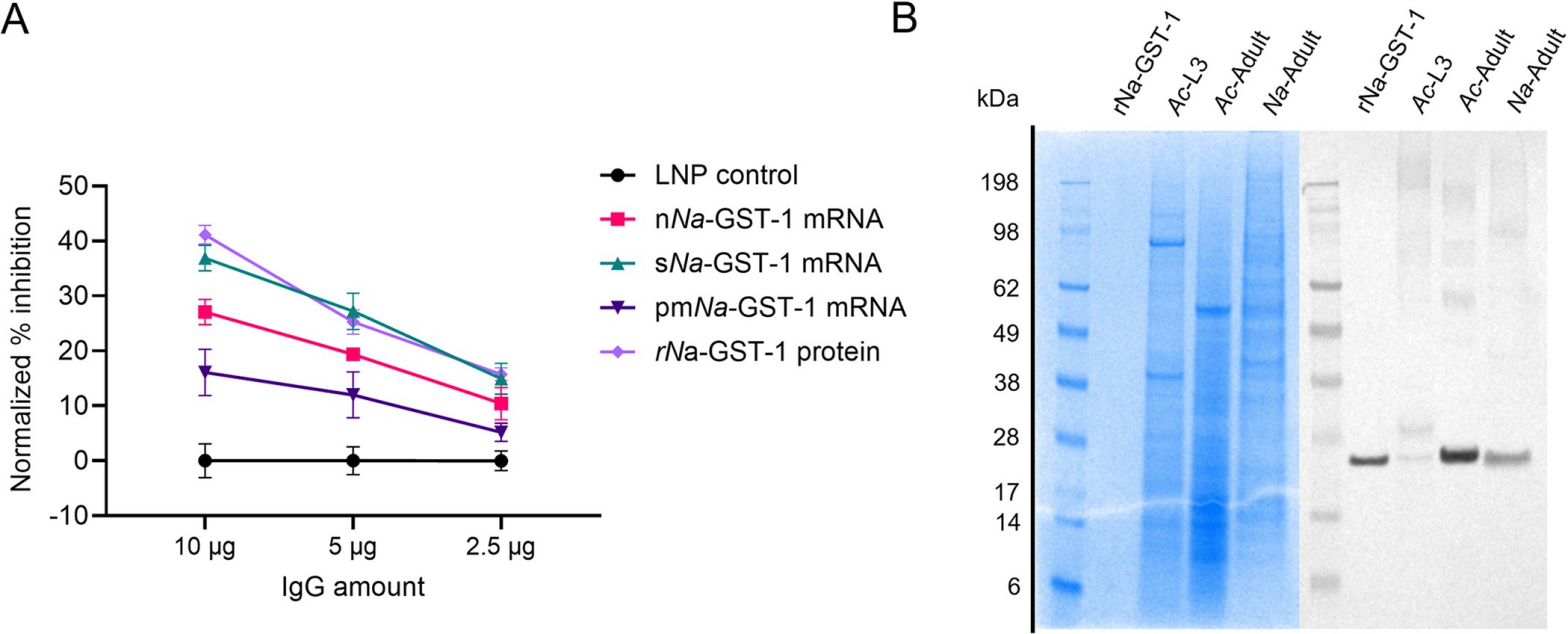
Neutralization of *Na*-GST-1 enzymatic activity after incubation with purified IgG from immunized mice. (A) Neutralization assay using multiple amounts of purified IgG. (B) Western blot analysis of pooled sera from the pm*Na*-GST-1 mRNA group against total proteins from L3 larvae and adult *A*. *caninum* and adult *N*. *americanus*.

After purification with protein G columns, the total IgG yield was similar across all mRNA groups, ranging from 1.8 to 2.1 mg/ml. Antibodies induced by s*Na*-GST-1 mRNA and r*Na*-GST-1 protein exhibited superior neutralization activity against *Na*-GST-1 compared to the other vaccine groups (Fig 4A). This suggests that the extracellular presence of *Na*-GST-1 as a free antigen enhances the likelihood of generating highly specific antibodies. Since antibodies induced by pm*Na*-GST-1 mRNA exhibited the lowest neutralization activity, it is possible that the GPI anchor impacts the availability of certain structural epitopes essential for neutralization of thiol transferase activity.

To confirm that mRNA vaccines induced antibodies capable of recognizing wild-type GST-1 proteins, we tested worm extracts from *Ancylostoma caninum* (L3 larvae and adults) and *Necator americanus* (adults) against pooled sera from the pmNa-GST-1 mRNA group. In line with the ELISA results for rNa-GST-1, antibodies detected wild-type GST-1 in all samples (Fig 4B). Cross-reactivity with *A*. *caninum* was expected due to the high identity and similarity of *Na*-GST-1 with GST-1 proteins from other hookworm species (S3 Fig).

## Discussion

Signal sequences, including signal peptides, transmembrane domains, and anchoring attachment sequences, among others, play a crucial role in guiding post-translational modifications and intracellular trafficking of proteins within living cells. In recombinant expression systems, removing or incorporating these sequences directly impacts these processes, altering the protein localization. By leveraging these aspects, the flexible design of RNA vaccines, while maintaining the same production pipeline, facilitates the creation of multiple variants of the same antigen, each with distinct intracellular distribution and, subsequently, exposure to the immune system. Through this approach, we successfully expressed *Na*-GST-1 in its native (mostly cytoplasmic), secreted, or membrane-anchored forms using recombinant mRNAs.

In these mRNA molecules, the incorporation of signal peptide sequences likely enhanced the translation efficiency of *Na*-GST-1 in mouse cells. Given that hookworms are extracellular parasites and have not undergone host-induced codon bias, the introduction of mammal-derived signal peptides was expected to be advantageous for translating a hookworm gene. Indeed, both pm*Na*-GST-1 and s*Na*-GST-1 seemed to be more highly expressed than n*Na*-GST-1 *in-vitro*, potentially influencing *in-vivo* results as well. However, antibody titers do not increase in direct proportion to expression levels, suggesting that spatial distribution—rather than translation efficiency alone—may play a more critical role in driving the humoral response. Additionally, native *Na*-GST-1, which mostly accumulated in the cytoplasm, elicited a stronger CD8+ T cell than extracellular *Na*-GST-1 (s*Na*-GST-1 and r*Na*-GST-1), compared to the LNP control. Altogether, these results indicate that spatial distribution plays a more crucial role in tailoring the immune response than mRNA translation efficiency. Our findings highlight the importance of understanding antigen localization before RNA vaccine design. Importantly, if a humoral response is the primary protective mechanism against a pathogen, extracellular antigen exposure increases humoral responses, a hallmark of subunit vaccines. Differently, if a cytotoxic T response is necessary, cytoplasmic accumulation of an antigen may enhance proteasome degradation and peptide loading into MHC-1.

The rationale for using *Na*-GST-1 as a vaccine antigen stems from its pivotal role in detoxifying harmful substances, widespread expression in hookworm tissues, and immunogenicity [22,52]. Significant attention has been directed to detoxification of heme, a byproduct of hemoglobin, within the gastrointestinal track [22,53,54]. Although *Na*-GST-1 activity has been detected in excretory/secretory products after *in-vitro* hookworm culture [55], it remains unproven how *Na*-GST-1 is secreted from the hookworm’s cells, given the absence of a classical signal peptide in its sequence. It is hypothesized that cytoplasmic GSTs utilize non-classical secretion pathways or unconventional mechanisms for secretion [56,57]. As previously suggested, *Na*-GST-1 could be excreted with conjugated molecules attached as a “molecular dispatch mechanism” or in exosome-like vesicles [55,57,58].

In the context of *Na*-GST-1 mRNA vaccines, antigen secretion or extracellular exposure enhanced antibody titers in mice. Previous preclinical studies with hamsters and r*Na*-GST-1 required a three-dose immunization schedule to boost antibody production [22]. Indeed, after a single immunization with r*Na*-GST-1 in mice, antigen-specific antibodies were not significantly detected, whereas notable titers were already achieved with pm*Na*-GST-1 and s*Na*-GST-1 mRNAs. While antibody levels with r*Na*-GST-1 increased after two doses, they remained inferior to those induced by pm*Na*-GST-1 and s*Na*-GST-1 mRNAs. Based on recently published data from the “Protection Associated with Rapid Immunity to SARS-CoV-2 (PARIS)” study, it is reasonable to expect that the observed antibody responses to the mRNA antigens may be long-lasting. The PARIS study showed that SARS-CoV-2 mRNA vaccination triggers a classical biphasic antibody decay, with an initial waning (like other vaccination platforms) followed by stabilization phase after 7 to 9 months [59].

Despite high antibody titers, pm*Na*-GST-1 mRNA generated the lowest proportion of antibodies that neutralized *Na*-GST-1’s thiol transferase activity. GPI-anchored proteins are located to the outer side of the cell membrane, with their orientation being influenced by the lipid environment. This could have impacted the exposure of certain epitopes or active sites, making them less accessible than in soluble forms of *Na*-GST-1. Circulating extracellular antigens (s*Na*-GST-1 and r*Na*-GST-1), on the other hand, induced the highest proportion of neutralizing antibodies, possibly due to more frequent direct encounters between them and naïve B cells. This direct binding, followed by antigen internalization and MHC-II presentation to T-helper cells, could have led to the production of antibodies with higher affinity. Since we only evaluated the thiol transferase activity of *Na-*GST-1, it is too early to draw definitive conclusions about neutralizing antibodies. Thus, while high levels of antigen-specific anti-GST-1 antibody are suspected to achieve protective immunity, we have not extended this observation to establish this aspect as a true correlate of protection. Future challenge experiments will be pivotal to determine if the *in vitro* observations correlate with *in vivo* efficacy outcomes.

The high genetic conservation of GST-1 across hookworm species highlights its potential as a broadly protective antigen. Our results show that antibodies induced by pm*Na*-GST-1 mRNA vaccine effectively recognized GST-1 in both L3 larvae and adult *A*. *caninum*. This cross-reactivity is important, as *N*. *americanus* and *A*. *caninum* share significant sequence identity and similarity with other medically important hookworm species, such as *A*. *duodenale* and *A*. *ceylanicum*. Given this conservation, the *Na*-GST-1-based vaccines has the potential to provide cross-species protection, targeting the primary hookworms affecting diverse geographic regions worldwide.

In the development of vaccines based on *Na*-GST-1, effective immunity relies not only on the generation of plasma cells to produce antigen-specific antibodies, but also on the establishment of memory cells that can quickly reestablish antibody production upon hookworm reinfection. Despite comparable antigen-specific antibody titers between s*Na*-GST-1 and pm*Na*-GST-1, the former did not induce significant counts of memory T cells relative to the LNP control. This observation suggests that immediate antibody levels induced by both vaccines may not be correlated with the specific subsets of T helper cells evaluated. Beyond these T cells results, s*Na*-GST-1 mostly reflected the immunoprofile of r*Na*-GST-1, except for the upregulation of IL-13. While antibody production was similar, pm*Na*-GST-1 may still outperform s*Na*-GST-1 in conferring enduring protection for stimulating higher counts of memory CD4+ subsets, which are pivotal in the expansion and differentiation of B cells. In healthy adults vaccinated with co-administered *Na*-GST-1 and *Na*-APR-1, CD4+ cells producing IL-2 and TNF were correlated with *Na*-GST-1 IgG levels [30]. Here, all vaccine candidates except s*Na*-GST-1 induced higher counts of memory effector CD4+ cells expressing IL-2, while TNF-α was up-regulated by pm*Na*-GST-1 in memory effector CD4+ T cells and by pm*Na*-GST-1 and r*Na*-GST-1 in memory central CD4+ T cells. Nevertheless, considering both antibody titers and thiol-transferase neutralization, s*Na*-GST-1 outperformed the other mRNA vaccine candidates under our study conditions.

These strategies for enhancing antigen exposure, as demonstrated with Na-GST-1, have been partially applied to other antigens, tailored to the pathogen and desired immune response. In contrast to our study, which assessed both surface exposure and cytoplasmic accumulation, most studies have focused primarily on surface-localized antigens. For example, studies on MERS-CoV mRNA vaccines found that the membrane-bound spike protein elicited stronger neutralizing antibodies than its secreted form, aligning with its native membrane-bound structure [60]. Similarly, the malaria transmission-blocking antigen Pfs25, anchored by a GPI or transmembrane domain, generated a more robust immune response compared to its secreted form [35]. These findings collectively highlight the significant role of antigen localization in modulating immunogenicity.

## Conclusion

r*Na*-GST-1, as a subunit vaccine, has proven to elicit strong immune responses and protection in preclinical hookworm animal models, as well as safety and immunogenicity in early clinical studies. The development of RNA vaccines based on *Na*-GST-1 represents an enticing alternative to subunit r*Na*-GST-1. In this study, we showed that the mRNA vaccine platform allows for the reevaluation of established antigens by manipulating their cellular spatial distribution, thereby modulating the immune response. Our findings reinforce that antigen localization is directly linked to the activation of certain immunological markers relevant to inducing protection against targeted pathogens. In this context, nucleic acid-based vaccines offer advantages by enabling precise manipulation of antigen expression and localization.

## Supporting information

S1 Fig Percentage of memory effector and memory central cytokine-producing CD4+ T cells after splenocyte restimulation with r*Na*-GST-1 protein. Statistical analysis was performed using the Kruskal-Wallis test, followed by Dunn’s test for pairwise comparison of groups. *p < 0.05, ** p < 0.01, ***p < 0.001.
(PDF)

S2 Fig Percentage of memory effector and memory central cytokine-producing CD8+ T cells after splenocyte restimulation with r*Na*-GST-1 protein. Statistical analysis was performed using the Kruskal-Wallis test, followed by Dunn’s test for pairwise comparison of groups. *p < 0.05, ** p < 0.01, ***p < 0.001, ****p < 0.0001.
(PDF)

S3 Fig Amino acid alignment and distance matrices of GST-1 proteins from various hookworm species. (A) Multiple sequence alignment generated using Clustal Omega implemented in Geneious Software, with conserved amino acids represented as dots. (B) Matrices showing the percentage (%) of identical and similar amino acids between sequences.
(PDF)
