# Supplementary figures and images for: Altering the intracellular trafficking of Necator americanus GST-1 antigen yields novel hookworm mRNA vaccine candidates
Source: PLoS Negl Trop Dis. 2025 Jan 10;19(1):e0012809. doi: 10.1371/journal.pntd.0012809 (PMC11756802; doi:10.1371/journal.pntd.0012809)

Th1

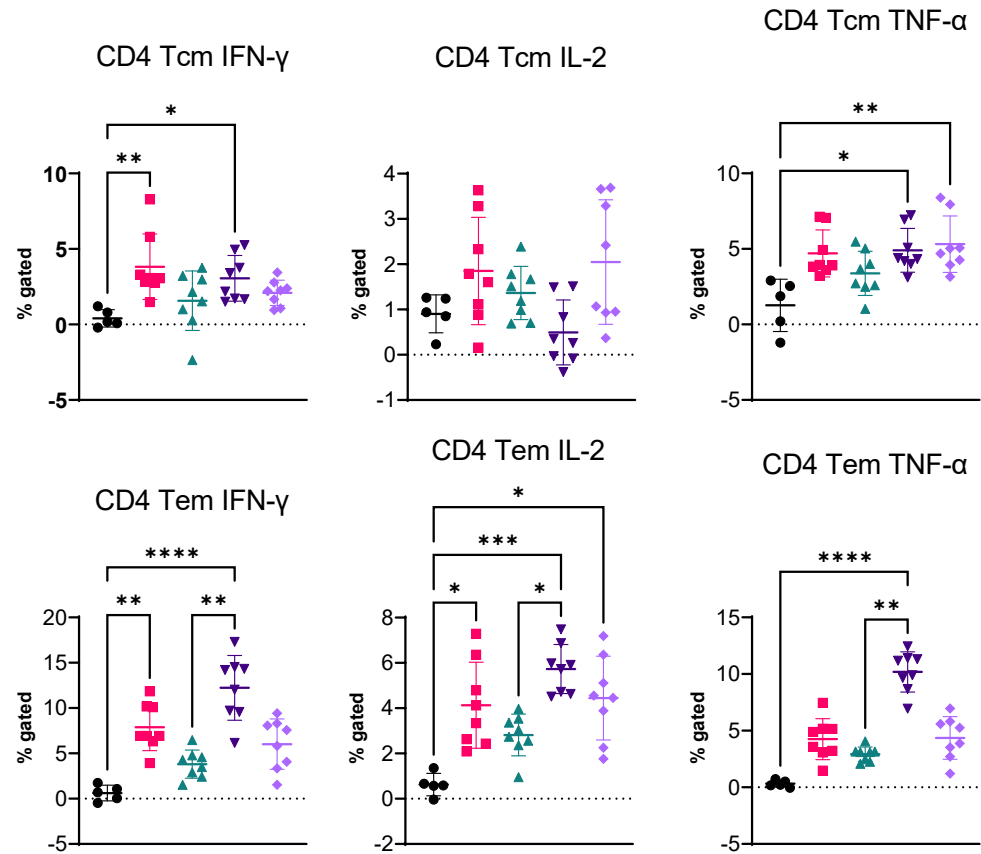

Th2

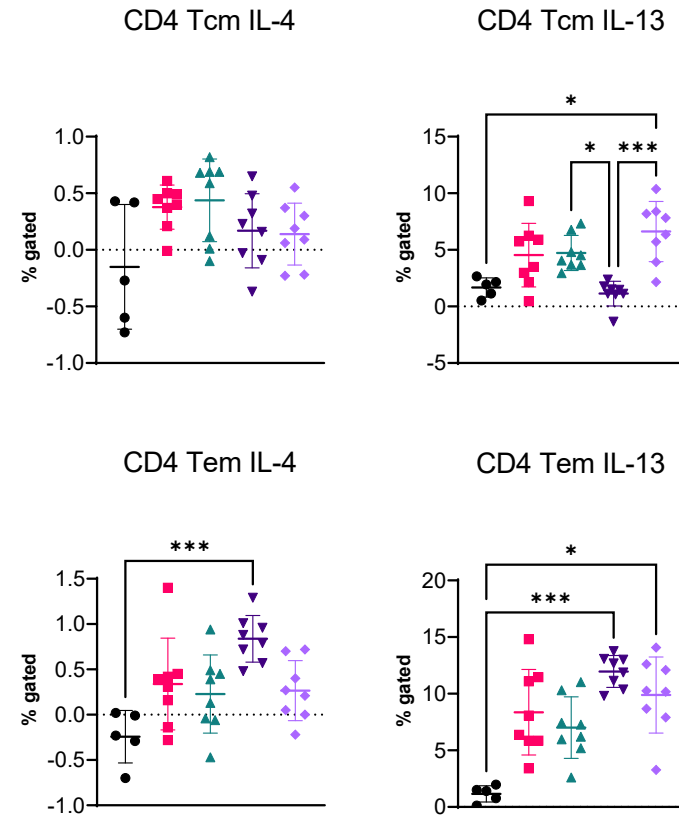

- LNP control
- nNa-GST-1 mRNA
- ▲ sNa-GST-1 mRNA
- ▼ pmNa-GST-1 mRNA
- rNa-GST-1 protein

S1 Fig

Supplement: S1 Fig — Statistical analysis was performed using the Kruskal-Wallis test, followed by Dunn’s test for pairwise comparison of groups. *p < 0.05, ** p < 0.01, ***p < 0.001. (PDF) [file pntd.0012809.s001.pdf]

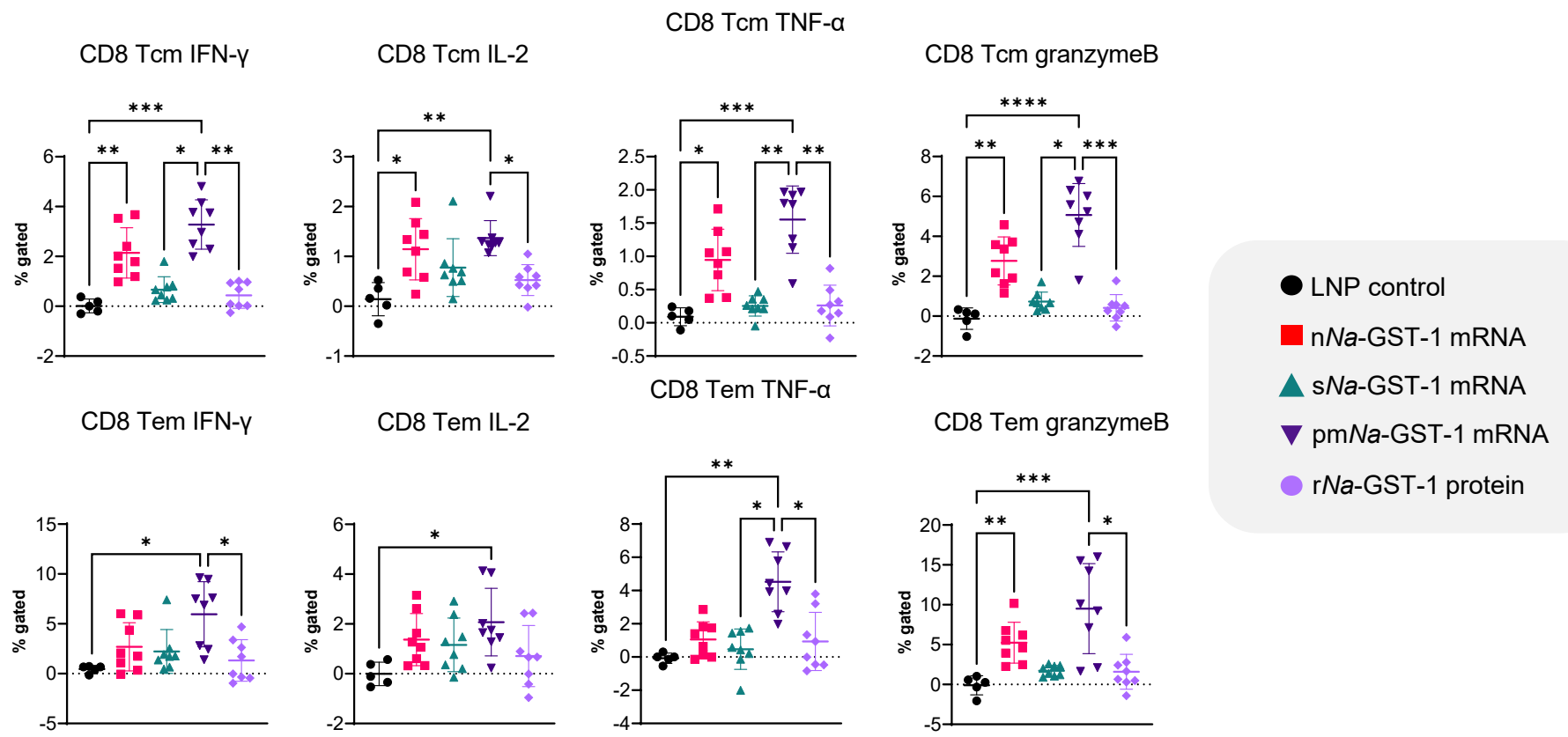

S2 Fig

Supplement: S2 Fig — Statistical analysis was performed using the Kruskal-Wallis test, followed by Dunn’s test for pairwise comparison of groups. *p < 0.05, ** p < 0.01, ***p < 0.001, ****p < 0.0001. (PDF) [file pntd.0012809.s002.pdf]
