# Supplementary material for: Altering the intracellular trafficking of Necator americanus GST-1 antigen yields novel hookworm mRNA vaccine candidates
Source: PLoS Negl Trop Dis. 2025 Jan 10;19(1):e0012809. doi: 10.1371/journal.pntd.0012809 (PMC11756802; doi:10.1371/journal.pntd.0012809)
Supplement: S3 Fig — (A) Multiple sequence alignment generated using Clustal Omega implemented in Geneious Software, with conserved amino acids represented as dots. (B) Matrices showing the percentage (%) of identical and similar amino acids between sequences. (PDF) [file pntd.0012809.s003.pdf]

A

```
Consensus                MVHYKLTYFDIRGAGECARQIFALAGQEFEDXRLTKEXFAPXKPKXPFGQVPVLEVDGKQ   60
N. americanus |FJ711440| .........A...............D.....V..D..Q..KV..DL..............   60
A. duodenale|KIH55339.1| ......S..........V.............N.....D...L...L..............   60
A. ceylanicum|EYC01075.1| .........PV....................V.....Q...V..NM....L.........   60
A. caninum|RCN49312.1|   ....................VL.........N.VA..D..AL...M....I.........   60

Consensus                LAQSLAICRYLARQFGXAGKSPFDEAVVDSLADQXSDXRVEIKPYFYTAIGMREGDLEQL  120
N. americanus |FJ711440| ................F....T............Y..Y.....SF...V.......V...  120
A. duodenale|KIH55339.1| .G.............DL.................HA.F......................  120
A. ceylanicum|EYC01075.1| .......S........F...T.....L.......Y..Y.....T.....V..MQ..KD..  120
A. caninum|RCN49312.1|   ................L.................H..F......................  120

Consensus                KKEVLLPAREKFFGFJTKFLKXNPSGFLVGDSVTWVDXLISEXIATXLXFXPEXLDGYPE  180
N. americanus |FJ711440| .........D.....I.....KS.........L....L.V..HN..M.T.V..F.E....  180
A. duodenale|KIH55339.1| .............VIL.....Q.............A.VV...S...L.S.V..L......  180
A. ceylanicum|EYC01075.1| ..D.........L..I.....K.S.............L....HC..M.TVA.DF......  180
A. caninum|RCN49312.1|   ........C......L...F.Q...............V....S...L.S.A..L......  180

Consensus                VKAHMEKVRAIPNLKKWIEXXPXRPF                                   206
N. americanus |FJ711440| ..E....I....K......TR.ETL.                                   206
A. duodenale|KIH55339.1| .........T.........NS.V...                                   206
A. ceylanicum|EYC01075.1| ...................TR.SST.                                   206
A. caninum|RCN49312.1|   ...................NS.A...                                   206
```

B

Identity

	1	2	3	4
1. *N. americanus* |FJ711440|		82	78	77
2. *A. ceylanicum*|EYC01075|	82		79	76
3. *A. caninum*|RCN49312|	78	79		90
4. *A. duodenale*|KIH55339|	77	76	90	

Similarity (Blosum45 with threshold 0)

1. *N. americanus* |FJ711440|		94	93	92
2. *A. ceylanicum*|EYC01075|	94		93	93
3. *A. caninum*|RCN49312|	93	93		98
4. *A. duodenale*|KIH55339|	92	93	98	

S3 Fig
